# Vitamin D as a Regulator of the Biological Clock—Implications for Circadian–Metabolic Dysregulation

**DOI:** 10.3390/ijms27073243

**Published:** 2026-04-02

**Authors:** Milena Vesković, Nikola Šutulović, Emilija Djuric, Dragan Hrnčić, Aleksandra Rašić Marković, Olivera Stanojlović, Dušan Mladenović

**Affiliations:** 1 Institute of Pathophysiology “Ljubodrag Buba Mihailović”, University of Belgrade Faculty of Medicine, 11000 Belgrade, Serbia; 2 Institute of Medical Physiology “Richard Burian”, University of Belgrade Faculty of Medicine, 11000 Belgrade, Serbia

**Keywords:** circadian syndrome, vitamin D, clock genes, metabolic syndrome, sleep disorders, depression

## Abstract

Circadian disruption represents a global health issue associated with cardiometabolic diseases, sleep disturbances, and mood disorders, driven by a pathophysiological network including clock gene dysregulation and impaired melatonin synthesis. Vitamin D exerts pleiotropic effects on metabolic regulation, immune function, neurotransmission, and possibly circadian synchronization. Emerging evidence suggests that vitamin D and its hydroxyderivatives modulate clock gene expression, influence transcriptional regulators such as retinoic acid receptor-related orphan receptors and REV-ERBs, and interact with melatonin synthesis and signaling. Vitamin D deficiency has been associated with metabolic syndrome, impaired sleep quality, and depression. Although interventional studies yield heterogeneous results, higher vitamin D status may confer protective metabolic and neurobehavioral effects. This review summarizes current evidence on the role of vitamin D in circadian disruption and evaluates its potential therapeutic relevance in circadian–metabolic dysregulation.

## 1. Introduction

Circadian disruption represents a global health problem in the 21st century affecting the majority of the world population. All humans are exposed to some form of circadian dysregulation due to a widespread use of artificial light sources which perturb the darkness required for melatonin synthesis. Sleep disorders and impaired sleep quality in depression, anxiety, obstructive sleep apnea, somatic diseases (asthma, chronic obstructive pulmonary disease, chronic pain, Parkinson’s disease, Alzheimer’s disease) further increase the burden of circadian disruption in human population [1,2].

Circadian misalignment has been unequivocally recognized as a risk factor for cardiometabolic diseases. It has been estimated that approximately 38,000 deaths in the USA are related to heart disease due to sleep disorder [3]. Inadequate sleep increases the risk of insulin resistance and metabolic syndrome; hence their co-appearance led to the designation of circadian syndrome [4,5]. The concept of “circadian syndrome” has been proposed as an extension of metabolic syndrome, originally defined by Zimmet et al. [6] by incorporating circadian disruption as a central component. The term was introduced by Shi et al. who actually expanded the cluster of cardiometabolic risk factors to include sleep disturbances and depression [7,8]. However, circadian syndrome remains relatively recent and not yet a universally adopted concept. Circadian syndrome includes the presence of sleep disturbances, depression, hypertension, hypertriglyceridemia, low high-density lipoprotein (HDL) cholesterol, glucose intolerance or type 2 diabetes mellitus, and metabolic dysfunction-associated steatotic liver disease (MASLD) due to circadian misalignment [8]. Common mechanisms in the pathogenesis of all features of circadian syndrome include dysregulation of clock genes and reduced melatonin synthesis which disturb metabolic homeostasis in the body along with sleep quality and emotional tone [9,10]. The role of circadian misalignment in the development of cardiometabolic diseases has been most clearly demonstrated in mice fed with a high-fat diet. Restriction of food intake only to the active phase prevents the development of obesity that otherwise would develop in mice on an isocaloric diet that is available during the whole day [10,11].

**Figure 1:**
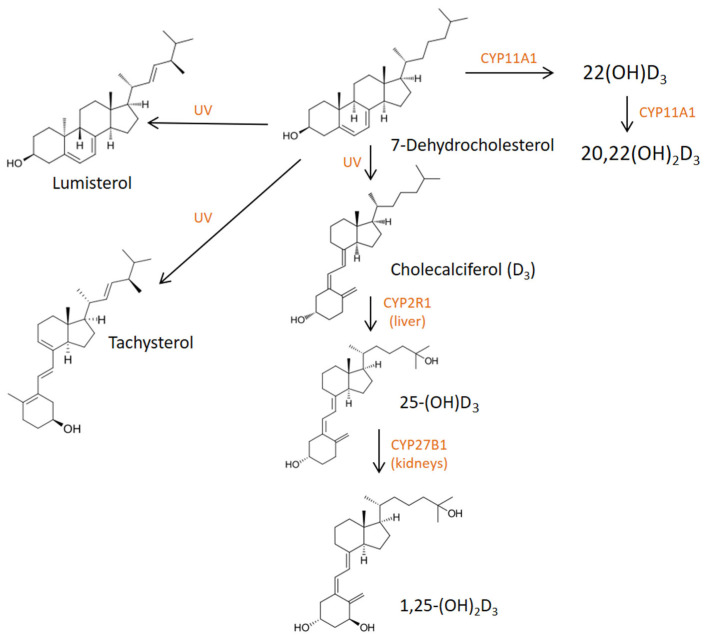
Vitamin D metabolism: formation of metabolites and hydroxyderivatives.

Since circadian misalignment is inevitable to some extent in the modern world, there is a need for the search of bioactive compounds that could reduce the risk of circadian syndrome. One of candidates is vitamin D. Vitamin D_3_ (cholecalciferol) is synthesized in the skin from 7-dehydrocholesterol by UV light and then transported to the liver where it is hydroxylated by 25-hydroxylase. 25-OHD_3_ is the most abundant form of vitamin D in the blood which is then hydroxylated in the kidneys into the active form calcitriol (1,25-(OH)_2_D_3_) by 1α-hydroxylase. Alternatively 25-OHD_3_ may be converted in kidneys into an inactive metabolite 24,25-(OH)_2_D_3_ [12]. The enzyme 1α-hydroxylase is induced by parathyroid hormone (PTH), while it is inhibited by hyperphosphatemia, fibroblast growth factor 23 (FGF-23), and 1,25-(OH)_2_D_3_ itself [13]. Apart from kidneys, 1,25-(OH)_2_D_3_ may be synthesized in macrophages due to Toll-like receptor (TLR) activation and interferon gamma (IFN-γ) stimulation [14]. 7-dehydrocholesterol may be further converted into lumisterol (L_3_) and tachysterol (T_3_) by UV-B light in the skin and then vitamin D_3_, L_3_, and T_3_ may be hydroxylated by CYP11A1 into 20-hydroxy- and 22-hydroxyderivatives [15] (Figure 1).

Although the first identified role of vitamin D was the regulation of bone and mineral homeostasis, now it is known that it has a pleiotropic effect including the regulation of energy metabolism [16], modulation of neurotransmission [17,18], regulation of insulin secretion and signaling [19,20], an immunomodulatory role [14], and possibly may synchronize the circadian clock [15]. Since it is fat-soluble, vitamin D is stored in adipose tissue and may regulate fat accumulation and adipokine and cytokine secretion [21]. The vitamin D receptor is expressed in more than a half of 400 human tissues and cells, including bones, kidneys, gut, brain, liver, skin, endothelial cells, adipose tissue, and immune cells [22]. The discovery of non-skeletal roles of vitamin D put this vitamin into the center stage for the possible prevention of metabolic syndrome and cardiovascular diseases. Based on this background, the aim of this narrative review was to summarize the current knowledge on the role of vitamin D on the course of circadian syndrome and its features, including metabolic syndrome, sleep quality, and depression, and to provide a critical explanation of the possible efficacy of vitamin D supplementation in the treatment of circadian syndrome. Additionally, the role of the circadian clock in the regulation of metabolism will be briefly summarized.

## 2. Circadian Clock and Metabolic Regulation

Circadian rhythm represents the approximately 24 h variations in physiological, biochemical, and behavioral processes that are entrained mainly by a light/dark cycle [23]. All cells contain molecular clocks organized in a hierarchical manner with the master clock located in the suprachiasmatic nucleus (SCN) in the hypothalamus [24]. Information on light exposure is transmitted to the SCN by retino-hypothalamic tract, a monosynaptic tract formed by axons derived from intrinsically photosensitive retinal ganglion cells that contain melanopsin [25]. SCN then synchronizes peripheral clocks via neural and hormonal pathways. Peripheral clocks may be synchronized by other stimuli such as food intake and physical activity, leading to the misalignment between central and peripheral clocks [23,26].

The molecular machinery of the circadian clock consists of transcriptional–translational feedback loops that include time-specific expression and interactions between transcriptional activators, repressors, and enzymes. Central transcriptional activators in the clock machinery are circadian locomotor output cycles kaput (CLOCK) and brain and muscle ARNT-like protein 1 (BMAL1) which heterodimerize and regulate the expression of about 43% of protein-coding genes [27,28]. The number of genes that undergo circadian variations, also known as clock-controlled genes, differs in different organs from 16% in the liver to 3% in the hypothalamus [28]. A study in baboons suggests that even 80% of protein-coding genes show diurnal variations in the expression which are responsible for metabolic and functional cycling during the day [29]. Apart from CLOCK-BMAL1 heterodimers, BMAL1 can also dimerize with neuronal PAS containing protein 2 (NPAS2) [3]. Photic stimuli increase BMAL1 expression and initiate the cycle of transcription–translation feedback loops that control circadian rhythm [30]. Among others both CLOCK-BMAL1 and BMAL1-NPAS2 complexes stimulate the expression of *Period* (*Per* 1, 2, 3) and *Cryptochrome* (*Cry* 1, 2) genes which are a part of the negative feedback loop. When present in sufficient amounts PER and CRY proteins repress CLOCK and BMAL1 activity early by the “displacement” type of repression and in the late phase by “blocking” repression [23,31]. In the “displacement” type of repression PER-CRY complex binds casein kinase 1δ (CK-1δ) in the cytosol and recruits CK-1δ to the nucleus where it phosphorylates CLOCK leading to the displacement of CLOCK-BMAL1 complex from the E-box DNA. On the other side, “blocking” repression includes PER degradation which releases CRY1, enabling the blocking of the transcriptional activity of CLOCK-BMAL1 complex without its displacement from DNA. Ultimately CRY1 is also degraded leading to the release of CLOCK-BMAL1 from repression, which initiates a new cycle of gene expression [23]. The secondary feedback loop includes another set of transcriptional regulators such as REV-ERBs and retinoic acid receptor-related orphan receptors (RORs) which are also induced by CLOCK-BMAL1 complex. REV-ERBs repress while RORs activate BMAL1 transcription by binding to retinoic acid receptor-related Orphan Receptor Element (RORE) [27].

Clock gene expression is further regulated at the post-transcriptional, post-translational, and degradation level to form a complex regulatory mechanism involved in a precise daily regulation of physiological processes according to environmental cues. Environmental cues (light/dark, food intake, physical activity) regulate the phase, amplitude, and period of clock protein expression via various regulators, including CK1 [23], FBXW7 [32], FBXL3 [33], O-GlcNAc transferase [34], sirtuins [35], phosphoprotein phosphatase [36], and adenosine monophosphate-activated protein kinase (AMPK) [37]. Circadian clock machinery regulates metabolism and inflammation, two major processes involved in the development of circadian syndrome. Food intake is also synchronized with daily activities due to connections between SCN and arcuate nucleus [38].

BMAL1 in association with CLOCK and NPAS2 regulates metabolism in a circadian manner also by the up-regulation of various transcription factors, including peroxisome proliferator-activated receptor alpha (PPARα) [39], SHP [40], paired box protein 4 (PAX4) [41], D-box binding PAR BZIP transcription factor (DBP) [42], and GATA binding protein 4 (GATA4) [43]. Through the interaction with PPARα, clock genes regulate daily variations in fatty acid oxidation, cholesterol synthesis, and ketogenesis in the liver [39,44]. It has been found that clock-controlled genes in liver lipid metabolism include acyl-CoA thioesterase, FAS, and 3-hydroxy-3-methylglutaryl CoA (HMG-CoA) reductase [45]. PPARα-deficient mice display attenuated circadian oscillations of sterol regulatory element-binding proteins (SREBPs), FAS, and HMG-CoA reductase expressions [46]. Regulatory interactions between PPARα and clock genes are bidirectional, since PPARα also regulates BMAL1 and REV-ERBα expression adding to the complex interactions between circadian misalignment and metabolic alterations [47]. SHP is an orphan receptor also involved in the regulation of lipid and bile acid metabolism which functions mainly as a transcription repressor [48]. Studies in *Shp^−/−^* mice have revealed that SHP increases fatty acid synthesis and reduces their oxidation through increased expression of acetyl CoA carboxylase (ACC) and PPARγ and reduced expression of PPARα [40]. Further research has found that SHP represses *Npas2* transcription via interactions with RORγ and REV-ERBα [40]. BMAL1 reduces glucose absorption via sodium glucose transporter 1 (SGLT1) by increased expression of PAX4 which binds to *Sglt1* promoter and represses *Sglt1* transcription [41]. GATA4 in hepatocytes increases cholesterol efflux into the bile via increased expression of ATP-binding cassette protein G5 (ABCG5) and ABCG8 thus contributing to the reduction in blood cholesterol level. BMAL1 up-regulates GATA4 expression in the liver stimulating cholesterol secretion into the bile [43].

Melatonin, a pineal gland hormone synthesized in the dark, may also mediate metabolic effects of a circadian clock. Darkness leads to the inhibition of GABAergic neurons in SCN with subsequent disinhibition of the paraventricular nucleus and activation of sympathetic fibers that promote melatonin synthesis [49]. The immediate effect of melatonin includes inhibition of insulin secretion by reduced cyclic adenosine monophosphate (cAMP) synthesis, a purposeful reaction during the night [50]. This leads to the development of cAMP signaling pathway hypersensitivity in the morning hours which increases the response of beta cells to incretins preparing Langerhans islets for the morning meal [51]. Circadian disruption with subsequent dysregulated melatonin synthesis contributes to insulin resistance and impaired insulin secretion leading to type 2 diabetes [52]. Additionally, melatonin exerts anti-inflammatory and antioxidant effects which are also diminished in circadian misalignment contributing further to insulin resistance [53].

## 3. The Role of Vitamin D in the Regulation of Circadian Rhythm

**Figure 2:**
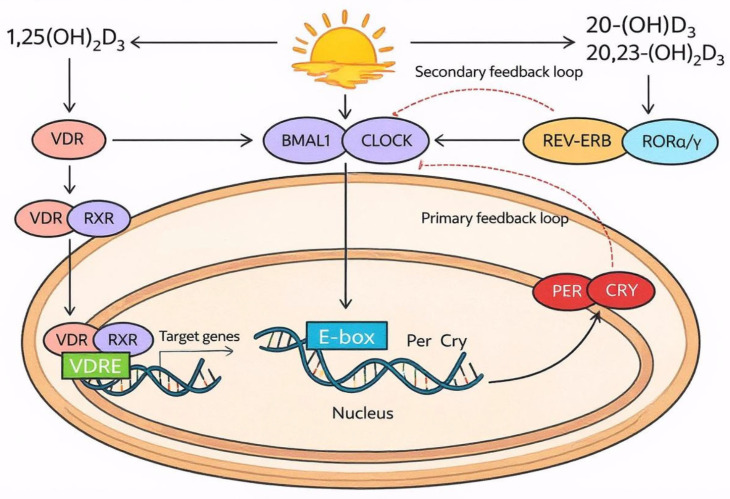
The role of vitamin D in the regulation of the circadian clock. Vitamin D receptor (VDR) interacts with CLOCK modulating the CLOCK-BMAL1 complex activity. Additionally 20-(OH)D_3_ and 20,23-(OH)_2_D_3_ act as inverse agonists of RORα/γ, thus inhibiting the secondary feedback loop in the clock mechanism. Through these actions vitamin D resets the phase and amplitude of transcriptional–translational feedback loops in the circadian clock. Black arrows—stimulation; Red arrows—inhibition. (Image created in PowerPoint and stylized using Canva, website/tool: Canva. (2026)).

It is well known that circadian disruption may result in decreased vitamin D synthesis due to reduced sun exposure and this contributes to the development of diseases [54,55]. However, the role of vitamin D in the regulation of the circadian clock mechanism is still not completely established. Ndiaye et al. [56] have postulated that vitamin D represents one of the molecules that links circadian disruption and oxidative stress in the skin. Additionally, 1,25(OH)_2_D_3_ was found to synchronize clock gene expression in adipose-derived stem cells [57]. However, this conclusion was based on qRT-PCR findings which analyzed expression only at the transcriptional level without appreciating post-transcriptional and post-translational modifications that regulate the phase of a circadian clock. A retrospective study suggests that vitamin D deficiency contributes to non-dipping hypertension, but this study did not provide any mechanism of this effect [58]. Although some studies claim that the relation between vitamin D and circadian disruption is bidirectional, current findings suggest that vitamin D may influence the major transcription factors in the regulatory loops by its hydroxyderivatives [15]. Apart from the classical activation pathway, vitamin D_3_ may undergo additional hydroxylation reactions producing alternative metabolites which also exert biological actions. CYP11A1 may catalyze further hydroxylation of these derivatives into dihydroxy- or trihydroxyderivatives which exert anti-inflammatory [59], antioxidant [60], antineoplastic [61,62], and antifibrogenic actions [63]. These vitamin D_3_ hydroxyderivatives, especially 20-hydroxyvitamin D_3_ and 20,23-dihydroxyvitamin D_3_, may affect the phase of the circadian clock as inverse agonists of RORα and RORγ and as agonists of aryl hydrocarbon receptors, while, potentially, they may also modulate the function of RORβ and REV-ERBs (Figure 2) [15]. Additionally, clock genes also regulate the synthesis of vitamin D_3_ hydroxyderivatives in the skin through the regulation of CYP11A1 activity [15]. However, a recent VitDHiD study in Kuopio, Finland, using a total monthly dose of 80,000 IU of vitamin D administered after breakfast and lunch revealed that *Per1* is a target gene of vitamin D [64,65]. Furthermore, a network analysis based on previous study determined 87 vitamin D target genes that undergo circadian variations in vivo, including transcription factors (CREB5, BCL6, MYC, RARA, JDP2 and FOSL2) and membrane receptors involved in the immune system regulation (CSF3R, CD3G, FCGR2A, C5AR1, CSF2RB, CCR7, PILRA, TREM1, FGR). Among genes regulated by vitamin D in a circadian manner are also genes involved in the metabolism and insulin sensitivity, but the response to vitamin D appears to be individually sensitive [66]. Vitamin D deficiency has been shown to be associated with reduced CLOCK and CRY2 expression in the liver at Zeitgeber time 1, while it is associated with reduced CLOCK and CRY1 expression and increased BMAL1 synthesis at the Zeitgeber time 13 [67]. Zeitgeber time is defined as time starting from the onset of the light phase. These effects clearly suggest that vitamin D, apart from its hydroxyderivatives, may regulate liver metabolism at least partly through its chronobiological actions.

The role of vitamin D in resetting the circadian clock has also been proposed in other organs, and one mechanism of its protective role in the skin photocarcinogenesis includes the modulation of clock gene expression. UV-B light causes a phase shift in clock gene expression with increased BMAL1 level in keratinocytes promoting the malignant transformation of skin cells, while vitamin D does not affect the phase and amplitude of BMAL1 expression, but reduces PER2 activity [68]. Topical vitamin D analog administration has also shown a therapeutic potential in psoriasis, but the therapeutic efficacy is dependent on circadian cycle phase when vitamin D is applied [69]. Vitamin D receptor was also found to interact with CLOCK in enterocytes and this interaction accelerates the binding of vitamin D receptor to response elements by enhancing histone acetylation. Circadian variations in vitamin D receptor-binding affinity for DNA causes oscillating calcium absorption in the small intestine during 24 h [70]. Vitamin D may also regulate the circadian rhythm in bones. Titanium-based biomaterials used as orthopedic and dental implants reduce PER1 expression and at the same time increase NPAS2 level in bone marrow-derived stem cells (BMSCs). Vitamin D deficiency reduces PER1 expression in BMSCs and interferes with titanium material implantation through the dysregulation of the circadian clock [71].

Vitamin D also interacts with melatonin in the regulation of the circadian rhythm. It has been shown that vitamin D regulates melatonin synthesis [15,72], while melatonin may exert some of its effects by binding to the vitamin D receptor [73]. During the evening and night a decrease in 1,25(OH)_2_D_3_ stimulates the activation of tryptophan hydroxylase 1 (TPH1) which converts serotonin to melatonin in the pineal gland [74]. In summary, all previous findings indicate that vitamin D, apart from its pleiotropic effects in various tissues, also exerts a chronobiological action and may influence the synchronization of the clock machinery with environmental cues.

## 4. The Relation Between Vitamin D and Circadian Disruption/Metabolic Syndrome

The information on the influence of vitamin D deficiency on the development of circadian syndrome are scarce and one reason may be a recent introduction of the term “circadian syndrome” in the scientific literature. However, the role of vitamin D deficiency in individual components of circadian syndrome has been more extensively studied.

**Figure 3:**
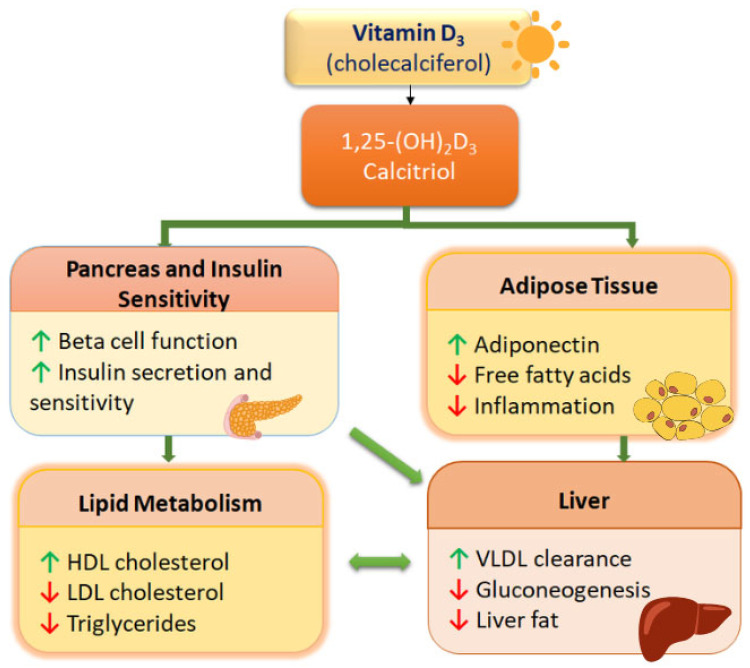
Effects of vitamin D on metabolic homeostasis. Calcitriol increases insulin secretion and tissue sensitivity to insulin both directly and indirectly by increased secretion of adiponectin and reduced inflammation. These changes contribute to antiatherogenic lipid metabolism and reduced fat accumulation in the liver. (Image created in PowerPoint and stylized using Canva, website/tool: Canva. (2026)).

It is well known that vitamin D reduces the risk of the development of metabolic syndrome. Accumulating evidence suggest that vitamin D serves as a regulator of immune responses and metabolic homeostasis, playing an important role in regulating insulin sensitivity, glucose and lipid metabolism. The identification of a vitamin D receptor and vitamin D-metabolizing enzymes in pancreatic β-cells, liver, skeletal muscle, adipocytes and immune cells provided a mechanistic basis for understanding its metabolic effects [75]. In pancreatic β-cells, the active form of vitamin D enhances insulin secretion through vitamin D receptor-dependent calcium flux, insulin gene transcription and proinsulin processing. In peripheral tissues, vitamin D improves insulin sensitivity by increasing the expression of insulin receptor and potentiating the downstream signaling cascade [20]. Activation of SIRT1/AMPK signaling promotes GLUT4 translocation, enhancing glucose uptake in skeletal muscle and adipose tissue [76]. Additional mechanism linking vitamin D to metabolic health is immune–metabolic crosstalk. In obesity, chronic low-grade inflammation impairs insulin signaling. In metabolic disorders, such as MASLD, numerous studies confirmed increase in proinflammatory and decrease in anti-inflammatory markers [77,78]. Vitamin D inhibits NF-κB and MAPK activation thus suppressing production of proinflammatory cytokines such as IL-1β, IL-6 and TNF-α. By reducing cytokines production, vitamin D limits inhibitory serine phosphorylation of IRS-1 and preserves insulin receptor signaling [79,80]. In adipose tissue, vitamin D exerts direct regulatory effects. Through vitamin D receptor signaling, it modulates adipocyte differentiation by regulation of Wnt/β-catenin pathways and restraining adipogenic transcription factors (PPARγ, C/EBPα) [81]. Adequate vitamin D signaling enhances adiponectin production, promoting fatty acid oxidation and insulin sensitivity, while deficiency is associated with reduced adiponectin and altered leptin signaling (Figure 3). Since vitamin D is fat-soluble and sequestered in adipose tissue, obesity further reduces the circulating 25-hydroxyvitamin D levels, creating a vicious cycle between vitamin D deficiency and metabolic dysfunction [82].

Recently it has been confirmed that vitamin D regulates metabolism in a light/dark phase-dependent manner. Metabolic genes that undergo circadian regulation by vitamin D include *IRS2*, *PFKFB3*, *PRKAG2*, *ASCL1*, *NAMPT*, *AQP9*, *SCL6A6*, and *SCL2A3* [66]. Insulin receptor substrate 2 (IRS2) is a signaling molecule activated by the insulin receptor and this indicates that vitamin D regulates insulin sensitivity in a circadian cycle. The *PFKFB3* gene encodes a bifunctional enzyme with 6-phosphofructo-2-kinase and fructose-2,6-bisphosphatase activity that regulates the level of fructose-2,6-bisphosphate, an allosteric activator of phosphofructokinase 1, a key glycolytic enzyme [83]. Genes *ASCL1* and *NAMPT* regulate fatty acid uptake and activation with CoA and NAD synthesis, respectively [84,85], while *SCL6A6*, *SCL2A3*, and *AQP9* facilitate taurine, glucose, and urea transport [86,87,88]. As previously described AMPK represents a central node in the regulation of energy metabolism and circadian rhythm through the phosphorylation of key metabolic enzymes and clock proteins and *PRKAG2* encodes the synthesis of AMPK subunit [66]. Vitamin D also regulates the activity of other transcription factors that are under the influence of clock genes including PPARα [89], SHP [90], PAX4 [91], and GATA4 [92] through the interactions between the vitamin D receptor and these factors. Hepatic *Shp* gene transcription is repressed by vitamin D and this modulates the circadian rhythm of bile acid synthesis [90]. These findings suggest that vitamin D may regulate metabolism through the entrainment of the circadian clock and provide the rationale for the chronobiological action of vitamin D in the prevention of metabolic syndrome.

Previous studies clearly demonstrated the role of vitamin D deficiency in sleep regulation and depression, although there are some controversies in results of previous studies. Vitamin D insufficiency/deficiency and sleep disturbances are bidirectionally linked; reduced blood vitamin D level may contribute to sleep disruption, while inadequate sleep and circadian disruption may reduce vitamin D intake and synthesis [93]. Massa et al. [94], have shown in a cross-sectional study that vitamin D deficiency is associated with short sleep (less than 5 h), reduced sleep efficiency, and increased wake time after sleep onset. Another experimental clinical study found that reduced vitamin D intake was associated with impaired sleep maintenance without the effects on sleep initiation and restorative quality of sleep [95]. A meta-analysis has unequivocally revealed that vitamin D deficiency with cut-off value of ≤20 ng/mL is associated with increased risk for sleep disorders especially with short sleep duration, poor sleep quality evident as frequent nocturnal awakenings, and sleepiness [96,97]. These findings have also been confirmed in children with more prevalent vitamin D deficiency in individuals with later weekday and weekend bedtimes [98,99]. A cross-sectional study in Japanese female university students revealed that increased loneliness score, greater social jetlag, and shorter sleep duration on weekdays accompanied vitamin D deficiency [100]. Additionally, vitamin D deficiency was found to be associated with sleep disturbances in pregnancy [101,102,103], although a study in pregnant women in Turkey did not confirm this effect [104].

Although the association between vitamin D deficiency and sleep disorders has been confirmed in a majority of research protocols, the studies investigating the effects of vitamin D supplementation on sleep quality are scarce and used a heterogenous methodological approach in different populations [105]. Musazadeh et al. [106] summarized in an interventional study that vitamin D supplementation improved sleep quality in the peripartum period and reduced the incidence of peripartum depression. Furthermore, supplementation with high doses of vitamin D (>4000 U/day) results in increased sleep efficiency and reduced sleep latency [107]. However, this effect may be variable in different individuals emphasizing the role of the personalized approach in the treatment of sleep disturbances with vitamin D [108]. The beneficial action of vitamin D on sleep may be augmented by the concomitant use of magnesium [108] and melatonin [109]. Optimization of vitamin D level was also found to improve sleep quality in dialysis patients [110]. In summary, meta-analyses and cross-sectional studies have found an association of vitamin D deficiency and impaired sleep quality, but interventional studies did not unequivocally confirm the beneficial role of vitamin D supplementation on the improvement of the sleep pattern.

The studies that investigated the association between vitamin D status and depression have shown contradictory results, but the majority of studies confirmed that depression was associated with vitamin D insufficiency/deficiency. Preclinical studies in rats have shown that vitamin D deficiency both in utero and after birth causes an increased brain volume with behavioral changes that mimic depressive-like state [111]. Additionally, the treatment of rats exposed to chronic low stress with vitamin D increased sucrose preference and stimulated object exploration indicating the alleviation of anhedonia and improvement of locomotor activity, two major features of depression. This effect was associated with increased dopamine transporter immunoreactivity in nc. accumbens even to a higher extent than after treatment with fluoxetine [112]. Beheshti et al. [113] confirmed the antidepressive effect of vitamin D in rats after nicotine withdrawal. Maternal vitamin D deficiency has been shown to promote depression in male offspring, the effect that can be reversed by vitamin D supplementation [114].

**Figure 4:**
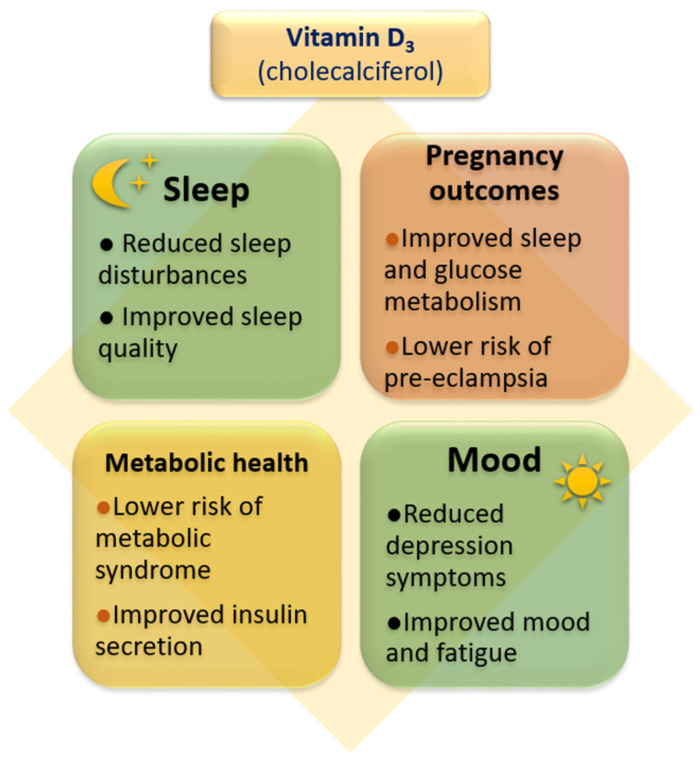
Diagram showing the major beneficial effects of vitamin D on sleep, mood and metabolic health, obtained from clinical studies.

Meta-analyses of randomized controlled trials in Canada, Australia, Iran, and Taiwan revealed an alleviation of depression after high-dose vitamin D treatment (>3500 IU/day) [115,116,117,118]. The advantage of these studies is a large number of participants (1203, 42,226, 1347, and 9840 participants, respectively). However, a research in a Spanish population found that 5250 IU/day vitamin D had no effect on depression course [119]. Meta-analyses of cohort studies mainly confirmed the increased risk of depression in vitamin D deficiency [120,121,122]. Only one meta-analysis interpreting the results in female population in Canada found that depression risk is independent on body vitamin D status. Wang et al. [123] revealed that vitamin D deficiency was associated with increased risk of postpartum depression, but had no effect on the development of depression during pregnancy. Studies in female population have suggested that the protective effect of vitamin D on depression may be more prominent in adolescents and young adult women than in postmenopausal women [124,125,126,127]. These findings may indicate that the role of vitamin D in the development of depression may be sex-dependent or related to age. Meta-analyses of cross-sectional studies unequivocally confirmed the reduced risk of depression in individuals with physiological vitamin D levels [120,121,122], although one of them [122] included a significantly lower number of participants than others. The reasons for the inconsistency between studies may include the different size of the studied population, heterogenous genetic background, sex, and age of participants, usage of different scales for depression diagnosis, various comorbidities in the studied populations, and different doses of vitamin D used in interventional studies. However, an umbrella meta-analysis of randomized controlled trials, cohort, and cross-sectional studies confirmed that physiological vitamin D level may reduce the risk of depression development especially in the population under age 50 [128]. Additionally, treatment with >5000 IU/day vitamin D for at least 20 weeks was found to be associated with more robust alleviation of depression symptoms. However, the protective role of vitamin D in the development of depression [129] cannot be excluded in the elderly population also [130]. A more recent meta-analysis concluded that vitamin D supplementation is beneficial in alleviating depression symptoms only if 25(OH)D_3_ is higher than 50 nmol/L (in the range of insufficiency), while no effect was observed under conditions of 25(OH)D_3_ concentrations below 50 nmol/L (in the range of deficiency) [131]. The list of clinical studies investigating the role of vitamin D in sleep disorders and depression as well as in circadian syndrome is summarized in Table 1, while major beneficial effects are shown in Figure 4.

Molecular mechanisms involved in the beneficial effect of vitamin D on sleep quality and depression include the regulation of circadian rhythm, modulation of neurotransmitter systems, immunomodulation, antioxidant activity, regulation of calcium ion homeostasis-associated proteins [108]. The vitamin D receptor is present in a wide variety of brain regions directly or indirectly involved in sleep regulation, including substantia nigra, lateral geniculate nucleus, hypothalamus, caudate nucleus, hippocampus, prefrontal cortex, and cingulate gyrus [99]. Among others, the vitamin D receptor is expressed in the SCN and regulates the expression of clock genes in association with a light/dark cycle. SCN contains two types of neurons which express vitamin D receptor: vasoactive intestinal polypeptide (VIP) and arginine vasopressin (AVP) neurons. VIP-expressing neurons located in the SCN core receive inputs from retino-hypothalamic tract and possess a long circadian rhythm, longer than 24 h. On the other side, neurons that express AVP are located in the shell region, possess circadian rhythm shorter than 24 h and they synchronize the activity of VIP-expressing neurons [132]. Vitamin D modulates the activity of both VIP and AVP neurons providing an impact on photic input from retina and synchronizing activity of AVP neurons via the complex regulation of the transcriptional and translational activity in the circadian network [108]. Additionally, vitamin D regulates *Period2* gene expression in microglia [133].

Vitamin D also modulates the key neurotransmitter systems involved in the regulation of sleep and the development of depression. Serotoninergic transmission is potentiated by vitamin D at the level of synthesis, degradation, and re-uptake of serotonin. Vitamin D stimulates serotonin synthesis in the brain by transcriptional activation of tryptophane hydroxylase 2 (*TPH2*) [134]. Additionally, vitamin D reduces the uptake and degradation of serotonin by down-regulating the expression of serotonin transporter and monoaminooxidase A (MAO-A) [135]. However, a meta-analysis did not confirm the rise in serotonin level in the brain after vitamin D supplementation [135]. Cholinergic transmission is also enhanced by vitamin D due to increased choline acetyltransferase activity and reduced acetylcholinesterase activity [136]. Dopamine level is increased in the striatum after vitamin D treatment [137], and the number of cultured rat dopaminergic neurons increases after incubation with vitamin D; this effect is probably mediated by glial-derived neurotrophic factor (GDNF) [138]. The protection of dopaminergic neurons by vitamin D may also be mediated by its antioxidant effect, evident as increased glutathione (GSH) level [139].

It is well known that depression and sleep disorders are associated with neuroinflammation and oxidative stress [140,141,142,143]. Vitamin D plays an immunomodulatory role with reducing the risk of at least some autoimmune diseases. 1,25-(OH)_2_D_3_ is synthesized in macrophages in response to TLR activation and FN-γ stimulation and it increases the microbicidal activity of macrophages with simultaneous decrease in TLR2 and TLR4 expression and proinflammatory cytokine production. At the same time vitamin D shifts the adaptive immunity towards Th2 response with the inhibition of Th17 and stimulation of regulatory T cells [144]. Insufficient sleep is associated with increased neuroinflammation, while vitamin D, after binding to vitamin D receptor, suppresses NF-κB activity in the brain, thus reducing neuroinflammation [145]. Vitamin D may regulate sleep and emotional tone through the up-regulation of brain-derived neurotrophic factor (BDNF) expression [146]. In chronic sleep deprivation vitamin D contributes to the maintenance of blood–brain barrier integrity by inhibition of matrix metalloproteinases and up-regulation of tight junction proteins [108].

Only one recent study in the United States performed within National Health and Nutrition Examination Survey (NHANES) revealed that vitamin D deficiency was associated with a 2.21-fold risk and vitamin D insufficiency with 33% increased risk for circadian syndrome [147]. Among the components of circadian syndrome the strongest association was found with short sleep. The major advantage of this study is a large number of participants, while the limitations include study design (a cross-sectional study) which cannot differentiate between cause and effect, inclusion of only US population, and reliance on self-reported data rather than on objective parameters.

**Table 1:** Clinical evidence on vitamin D and circadian syndrome. This table summarizes the key clinical studies investigating the relationship between vitamin D status, supplementation, and circadian syndrome-related outcomes.

Study Design	Number of Patients	Vitamin D Assessment/Treatment	Population	Major Findings	Reference
Cross-sectional	1455	Serum 25(OH)D levels	Adults (Middle East)	Vitamin D deficiency independently associated with higher prevalence of circadian syndrome (central obesity, hypertension, dyslipidemia, hyperglycemia, short sleep, depression).	[147]
Prospective cohort	3048	Serum 25(OH)D levels	Community-dwelling older men	Lower vitamin D associated with short sleep duration and poorer sleep efficiency measured by actigraphy.	[94]
Cross-sectional	141	Serum 25(OH)D levels	Hemodialysis patients	Vitamin D deficiency associated with greater sleep disturbance severity and poor sleep quality.	[101]
Cross-sectional	153	Serum 25(OH)D levels	Third-trimester pregnant women	Inadequate vitamin D associated with sleep deprivation and poorer sleep parameters.	[104]
Cross-sectional	890	Plasma 25(OH)D levels	Pregnant women (Singapore)	Vitamin D deficiency associated with poor sleep quality and night-time eating behavior.	[101]
Randomized controlled trial	169	50,000 IU vitamin D every 2 weeks for 8 weeks	Postpartum women	Supplementation significantly reduced postpartum depression and fatigue.	[126]
Randomized controlled trial	243	20,000 IU/week for 6 months	Adults with low vitamin D	No overall significant effect on depression score; subgroup effects observed.	[125]
Case series	54	Vitamin D supplementation (dose not standardized)	Depressed adolescents	Low baseline vitamin D; depressive symptoms improved after supplementation.	[124]
Cross-sectional	160	Serum 25(OH)D levels	Japanese female university students	Vitamin D deficiency associated with insomnia symptoms and social jetlag.	[100]

## 5. Conclusions and Future Directions

Vitamin D has a promising role as a regulator of both circadian rhythm and metabolic homeostasis and potentially in the era of intense light pollution. It acts at several levels of biological clock, through modulation of the REV-ERB/ROR feedback loop and direct regulation of PER gene expression. Additionally vitamin D regulates melatonin synthesis through the activation of TPH1 which converts serotonin to melatonin in evening hours, potentially influencing the sleep–wake cycle. It has been confirmed that vitamin D reduces the incidence of metabolic syndrome by increasing insulin sensitivity and insulin release directly via the interaction of vitamin D receptor with insulin signaling molecules and indirectly by reducing adipogenesis and synthesis of proinflammatory cytokines in adipose tissue. These processes overlap substantially with pathways implicated in both metabolic and circadian dysregulation.

Preclinical and clinical studies have shown that vitamin D deficiency is associated with depression, sleep disorders and reduced sleep quality, but the role of vitamin D supplementation in the prevention of depression and sleep disorders has not been confirmed unequivocally. The concept of circadian syndrome provides an integrative framework linking circadian disruption with metabolic and neurobehavioral outcomes. The term circadian syndrome has been recently introduced and for this reason, the data on the effects of vitamin D on circadian syndrome are still relatively scarce.

Future research should be oriented towards better understanding of the molecular mechanisms of vitamin D interactions with clock genes integrating molecular clock phase shifts with clinical outcomes. Additionally, the proper timing of vitamin D administration in its deficiency to maximize its effect should be further delineated in prospective studies. Also future research should be focused on the interactions between vitamin D and melatonin in the improvement of sleep quality and metabolic dysregulation. Additionally, the basis for differential individual susceptibility to vitamin D supplementation should be further established. These findings would further clarify the role of vitamin D in the therapy and prevention of circadian disruption-related metabolic syndrome.

## Data Availability

No new data were created or analyzed in this study. Data sharing is not applicable to this article.

## Abbreviations

The following abbreviations are used in this manuscript:

ACC	Acetyl-CoA Carboxylase
AMPK	Adenosine Monophosphate-Activated Protein Kinase
AQP9	Aquaporin 9
ASCL1	Achaete-Scute Family BHLH Transcription Factor 1
AVP	Arginine Vasopressin
ABCG5	ATP-Binding Cassette Subfamily G Member 5
ABCG8	ATP-Binding Cassette Subfamily G Member 8
BDNF	Brain-Derived Neurotrophic Factor
BMAL1	Brain and Muscle ARNT-Like 1
BMSCs	Bone Marrow-Derived Stem Cells
cAMP	Cyclic Adenosine Monophosphate
C/EBPα	CCAAT/Enhancer-Binding Protein Alpha
CLOCK	Circadian Locomotor Output Cycles Kaput
CREB	cAMP Response Element-Binding Protein
CRY	Cryptochrome
CSF2RB	Colony Stimulating Factor 2 Receptor Subunit Beta
CSF3R	Colony Stimulating Factor 3 Receptor
CYP11A1	Cytochrome P450 Family 11 Subfamily A Member 1
CK-1δ	Casein Kinase 1 Delta
DBP	D-Box Binding PAR BZIP Transcription Factor
E-box	Enhancer Box
FAS	Fatty Acid Synthase
FBXL3	F-Box and Leucine-Rich Repeat Protein 3
FBXW7	F-Box and WD Repeat Domain Containing 7
FCGR2A	Fc Gamma Receptor IIA
FGF-23	Fibroblast Growth Factor 23
FGR	FGR Proto-Oncogene, Src Family Tyrosine Kinase
GABA	Gamma-Aminobutyric Acid
GATA4	GATA Binding Protein 4
GDNF	Glial Cell-Derived Neurotrophic Factor
GLUT4	Glucose Transporter Type 4
GSH	Glutathione
HDL	High-Density Lipoprotein
HMG-CoA	3-Hydroxy-3-Methylglutaryl-Coenzyme A
IFN-γ	Interferon Gamma
IL-1β	Interleukin 1 Beta
IL-6	Interleukin 6
IRS-1	Insulin Receptor Substrate 1
IRS2	Insulin Receptor Substrate 2
L3	Lumisterol
MAO-A	Monoamine Oxidase A
MAPK	Mitogen-Activated Protein Kinase
MASLD	Metabolic Dysfunction-Associated Steatotic Liver Disease
NAD	Nicotinamide Adenine Dinucleotide (Oxidized Form)
NAMPT	Nicotinamide Phosphoribosyltransferase
NF-κB	Nuclear Factor Kappa B
NHANES	National Health and Nutrition Examination Survey
NPAS2	Neuronal PAS Domain Protein 2
O-GlcNAc	O-Linked N-Acetylglucosamine
PAX4	Paired Box Protein 4
PER	Period
PFKFB3	6-Phosphofructo-2-Kinase/Fructose-2,6-Bisphosphatase 3
PILRA	Paired Immunoglobulin-Like Type 2 Receptor Alpha
PPAR	Peroxisome Proliferator-Activated Receptor
PRKAG2	Protein Kinase AMP-Activated Non-Catalytic Subunit Gamma 2
PTH	Parathyroid Hormone
qRT-PCR	Quantitative Reverse Transcription Polymerase Chain Reaction
RORE	Retinoic Acid Receptor-Related Orphan Receptor Response Element
ROR	Retinoic Acid Receptor-Related Orphan Receptor
SCN	Suprachiasmatic Nucleus
SGLT1	Sodium-Glucose Cotransporter 1
SHP	Small Heterodimer Partner
SIRT1	Sirtuin 1
SREBP	Sterol Regulatory Element-Binding Protein
T3	Tachysterol
TLR	Toll-Like Receptor
TNF-α	Tumor Necrosis Factor Alpha
TPH	Tryptophan Hydroxylase
VIP	Vasoactive Intestinal Polypeptide
